# GNSS Timing Performance Assessment and Results Analysis

**DOI:** 10.3390/s22072486

**Published:** 2022-03-24

**Authors:** Lin Zhu, Huijun Zhang, Xiaohui Li, Feng Zhu, Yinhua Liu

**Affiliations:** 1 National Time Service Center, Chinese Academy of Sciences, East Shu Yuan Road, Xi’an 710600, China; zhj@ntsc.ac.cn (H.Z.); xiaohui@ntsc.ac.cn (X.L.); zhufeng@ntsc.ac.cn (F.Z.); liuyh@ntsc.ac.cn (Y.L.); 2 Key Laboratory of Precision Navigation and Timing Technology, Chinese Academy of Sciences, Xi’an 710600, China; 3 School of Astronomy and Space Science, University of Chinese Academy of Sciences, Yu Quan Road, Beijing 100049, China

**Keywords:** GNSS, timing performance, time transfer accuracy, assessment

## Abstract

Global Navigation Satellite System (GNSS) timing is a main service function. Each GNSS has its own time performance specification. However, a uniform timing performance assessment methodology and its outcomes do not exist. Firstly, the timing performance specifications of each GNSS are analyzed. Then, time transfer accuracy is considered as the key GNSS timing performance indicator. Secondly, an assessment method for the Coordinated Universal Time (UTC) published by the Bureau International des Poids et Mesures (BIPM) and UTC kept by the National Time Service Center of China (UTC(NTSC)) is proposed. Thirdly, the uncertainty budget of the assessment method is given. The timing performances of BDS, GPS, GLONASS, and Galileo are assessed and compared. The results show that the time transfer accuracy of BDS, GPS, GLONASS, and Galileo was 13.8 ns, 4.5 ns, 16.8 ns, and 4.2 ns, respectively, in 2021, meeting their performance requirements specified by GNSS (30 ns or 40 ns). Meanwhile, the assessment results of GPS and Galileo are much better than requirements, while the assessment results of BDS and GLONASS show fixed time offset and can still be improved further. If the local reference time of GNSS users can be connected with UTC, this assessment method can be used.

## 1. Introduction

Global Navigation Satellite Systems mainly include the Global Positioning System (GPS) of the United States, Global Navigation Satellite System (GLONASS) of Russia, the Global Satellite Navigation System (Galileo) of the European Union, and the BeiDou Navigation Satellite System (BDS) of China, which provide global users with services, such as positioning, velocity measurement, and timing.

Timing is a basic service provided by a GNSS to realize time transmission by GNSS signal in space (SIS). Its coverage is wide, the propagation attenuation is small, and it is not limited by the number of users. GNSS timing is widely used in the time synchronization of the military digital communication network, the telecommunication network, electricity, etc. [1,2]. With the rapid development of global integration, all industries around the world have become more dependent on high-precision GNSS timing. When GNSS timing services are abnormal, the global distribution industries of communications, transportation, finance, and others cannot achieve a unified time, which will have a huge impact. For example, from 12 July 2019 to 17 July 2019 of UTC, the Galileo system service was degraded and interrupted for nearly 117 h. The technical incident originated from an equipment malfunction in the Galileo ground infrastructure, affecting the calculation of time and orbit predictions that generate the navigation message. The malfunction affected different elements on the ground facilities. During this period, Galileo signals were unavailable, and users underwent services outage [3,4]. Therefore, it is very important to assess the GNSS timing performance to provide a reference for users while using time services.

With the development of GNSS, optimization and standardization of the GNSS timing performance by GNSS providers have gradually been carried out. Currently, GPS, GLONASS, Galileo, and BDS have published open service performance specifications [5,6,7]. However, the indicators of timing performance are different across GNSSs. In [8], timing accuracy was revealed as a key indicator of BDS timing performance, with a result better than 20 ns. In [7,9], the indicator of GPS and GLONASS timing performance was defined as a time transfer accuracy less than 30 ns and 40 ns, respectively. In [10], the performance of the Galileo time service, also called the time dissemination service, was indicated by a UTC time dissemination accuracy of less than 30 ns.

Performance reports of open service are published periodically by GNSS providers according to GNSS open service performance specification. Unfortunately, due to different timing performance indicators, the assessment results in reports are not comparable. In addition, GNSS timing is monitored and timing performances are assessed by international organizations and research institutions. For example, the International GNSS Service (IGS) set up a Multi-GNSS Experiment (MGEX) to track, collate, and analyze all available GNSS signals [11]. These products contain postprocessed monitoring results in real time, rather than service performance assessment results. The GNSS service performance analysis report provided quarterly by the William J. Hughes Technical Center of Federal Aviation Administration (FAA) only contains GPS and Galileo, and the timeliness is poor [12]. The International GNSS Monitoring and Assessment System (iGMAS) regularly publishes service performance assessment results for BDS, GPS, GLONASS, and Galileo, but no assessment results of timing performance [13]. The authors of [14] referred to the role of GNSS in disseminating time and presented the relations of UTC with predictions of UTC(k) disseminated only by GPS and GLONASS. They did not focus on the methodology of GNSS timing performance assessment or a comparison of time transfer accuracy assessment results across GPS/GLONASS/BDS/Galileo. At present, there is no uniform assessment methodology of timing performance and its outcomes.

In order to promote GNSS compatibility and interoperability, the International Committee on GNSS (ICG) established the Interoperability and Service Standards Subgroup and it has held regular workshops and seminars to discuss open service performance standards. At present, ICG has published the latest version of *Guidelines for Developing Global and Regional Navigation Satellite Systems Performance Standards* to provide GNSS users with unified GNSS timing performance standards [15,16,17]. For multi-GNSS users, it is necessary to clarify the timing performance indicators and assessment methods, which ultimately better guide the use of time services and the manufacture of timing equipment.

In this paper, the assessment of GNSS timing performance is investigated. The principle of GNSS timing is studied (Section 2.1). On this basis, the definition and connotation of the key indicators of GNSS timing performance are discussed. Additionally, an assessment method of GNSS timing performance is proposed on the basis of UTC(NTSC) and the offset between Rapid UTC (UTCr) and UTC(k) provided by BIPM (Section 2.2), and the various components of uncertainty are analyzed. Lastly, the assessment results of GNSS timing performance are compared and analyzed (Section 3).

## 2. The Key Indicators and Assessment Method of GNSS Timing Performance

### 2.1. Principle of GNSS Timing

Currently, UTC is the international standard timescale, which is obtained from an average of about 450 atomic clocks maintained in about 80 national timing laboratories around the world. The laboratories participate in the UTC calculation through sending clock data to BIPM and generate their real-time local estimates of UTC called UTC(k). UTC(k) estimates are contributions of UTC, where the “k” denotes the particular timing laboratory [18,19]. For example, UTC(USNO) is a contribution of UTC generated at the US Naval Observatory (USNO). UTC(SU) is a contribution of UTC generated at the Russian Metrological Institute of Technical Physics and Radio Engineering. UTC(NTSC) is a contribution of UTC generated at the National Time Service Center of China (NTSC). BIPM regularly publishes the difference between UTC and UTC(k) [20].

Each GNSS has an independent time system that generates its internal reference timescale, which is called the system timescale. The system timescales of GPS, GLONASS, BDS, and Galileo (GNSST) correspond to GPST, GLONASST, GST, and BDT respectively. The system times of BDS, GPS, and Galileo are accumulated continuously according to the International System of Unit (SI) of seconds from their individual start epoch [21,22,23]. GLONASST is periodically corrected with leap seconds [24]. GNSST is necessarily steered to its time base, i.e., the national UTC(k). Consequently, the traceability of GNSST to UTC(k) is accomplished by a precise connection between the GNSS control center and national UTC(k) laboratories. GPST is steered closely to UTC(USNO) modulo 1 s. GLONASST is steered to UTC(SU). GST is steered to a reference UTC time provided by the Galileo Time Service Provider (GTSP) according to contributions from European UTC(k) laboratories. BDT is related to the UTC through UTC(NTSC). The time offset between GNSST and UTC/UTC(k) called the UTC Offset (UTCO) is determined by a time comparison between the GNSS control center and national UTC(k) laboratories. The prediction model parameter of UTCO based on the measurement processing is regularly broadcast to users by GNSS satellite.

**Figure 1:**
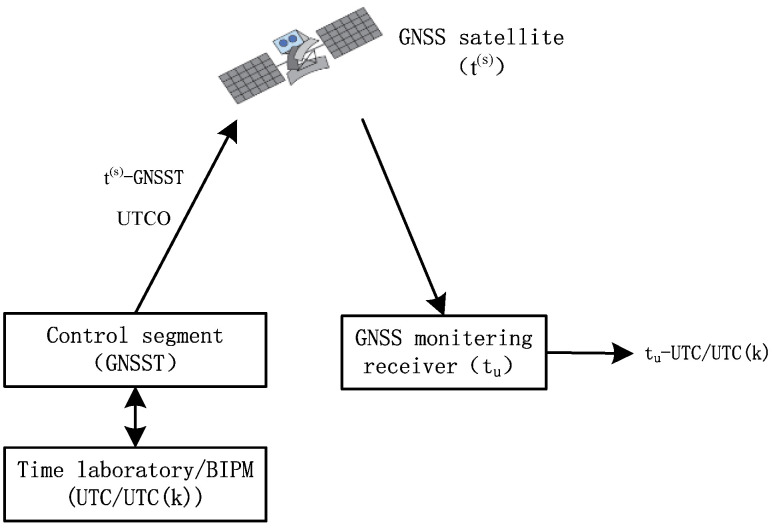
Principle of GNSS timing.

On the premise that the antenna coordinates of the GNSS monitoring receiver are known precisely, the time offset between the receiver time (t_u_) and system time (GNSST)) can be obtained from one visible satellite by subtracting various correction elements such as the geometric distance, satellite clock error, ionosphere delay, and troposphere delay from the pseudo-range observation. Furthermore, the time offset between receiver time and broadcast UTC, recorded as t_u_-UTC/UTC(k), is obtained using the broadcast UTCO model parameter. At this stage, GNSS timing is completed [25,26,27]. Usually, several satellites can be observed simultaneously at the receiver. Thus, the timing performance can be improved by weighted averaging of the receiver time offset from multiple satellites [27]. The principle of GNSS timing is illustrated in Figure 1.

As shown in Figure 1, t_u_-UTC/UTC(k) can be calculated as follows:

t_u_-UTC/UTC(k) = [t_u_ − t^(s)^]_Mon_ + [t^(s)^ − GNSST]_Nav_ + [UTCO]_Nav_, (1)

where the subscript Mon indicates that the time offset is obtained by receiving a SIS, and Nav indicates that the time offset is obtained from a navigation message.

### 2.2. Assessment Method of GNSS Time Transfer Accuracy

According to BDS open service performance specification, timing accuracy is a specified indicator to assess BDS timing performance. Timing accuracy refers to the 95% probability level of the differences between BDT determined by BDS and real BDT [8], while the open service performance specification of both GPS and GLONASS specifies the time transfer accuracy as the indicator of timing performance [7,9]. Time transfer accuracy refers to the 95% probability level of the instantaneous time transfer error sequence between the estimated receiver time offset from a reference realization of UTC and the true offset over a specified time interval [17]. According to GNSS timing principle and the definition of performance indicators, the time transfer accuracy is considered as the main indicator of GNSS timing performance. It is difficult to obtain the real UTC. In fact, it is difficult to obtain the real offset of receiver time with respect to UTC. Therefore, the estimated receiver time offset, recorded as t_u_-UTC_GNSS_, refers to the offset between receiver time and UTC that GNSS broadcasts; the real receiver time offset, recorded as t_u_-UTC_true_, refers to the offset between receiver time and ‘real UTC/UTC(k)’, and the time transfer error sequence is recorded as UTC_GNSS_-UTC_true_.

#### 2.2.1. Assessment Principle of GNSS Time Transfer Accuracy

**Figure 2:**
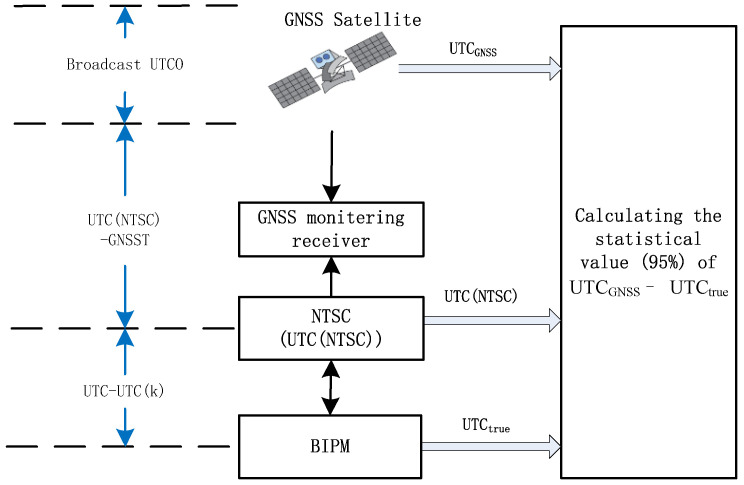
Principle of GNSS time transfer accuracy assessment.

The assessment principle of GNSS time transfer accuracy which takes UTC as the reference timescale and UTC(NTSC) as the intermediate timescale is shown in Figure 2. Firstly, receiver time is synchronized to UTC(NTSC). Secondly, GNSS time transfer error sequences are obtained as follows: (1) the time offset UTC(NTSC)-UTC_GNSS_ is estimated by data processing which follows the GNSS timing principle described in Section 2.1; (2) the real-time offset UTC(NTSC)-UTC_true_ is calculated using offset UTC-UTC(k) from BIPM; (3) then, the offset UTC(NTSC)-UTC_GNSS_ is converted to UTC_GNSS_-UTC_true_. Finally, the GNSS time transfer accuracy can be obtained by calculating the 95% probability level of GNSS time transfer error sequence. For BDS, GPS, GLONASS, and Galileo, UTC_true_ refers to UTC, UTC(USNO), UTC(SU), and UTC, respectively. UTC_GNSS_-UTC_true_ offsets of BDS, GPS, GLONASS and Galileo are calculated according to Equations (2)–(5).

UTC_BDS_-UTC = −[UTC(NTSC)-UTC_BDS_]_Mon_ − [UTC-UTC(NTSC)]_BIPM_, (2)

UTC_GPS_-UTC(USNO) = −[UTC(NTSC)-UTC_GPS_]_Mon_ − [UTC-UTC(NTSC)]_BIPM_ + [UTC-UTC(USNO)]_BIPM_, (3)

UTC_GLONASS_-UTC(SU) = −[UTC(NTSC)-UTC_GLONASS_]_Mon_ − [UTC-UTC(NTSC)]_BIPM_ + [UTC-UTC(SU)]_BIPM_, (4)

UTC_Galileo_-UTC = −[UTC(NTSC)-UTC_Galileo_]_Mon_ − [UTC-UTC(NTSC)]_BIPM_. (5)

#### 2.2.2. The Key Technology of GNSS Time Transfer Accuracy Assessment

The key technology of GNSS time transfer accuracy assessment is to obtain a high-precision time offset of UTC(NTSC)-UTC_GNSS_ through a GNSS monitoring receiver connected to UTC(NTSC). The GNSS monitoring receiver is the core equipment for obtaining UTC(NTSC)-UTC_GNSS_. Additionally, the receiver delay calibration and receiver clock control strategy are crucial.

The receiver delay refers to the delay from phase center of antenna to one pulse per second (1 PPS) reference signal input port. It is divided into two parts: one part is from the RF input port to the internal pseudo-range measurement latch point, and the other is from the latch point to the 1 PPS reference signal input port. It is also necessary to consider the inter-frequency bias (IFB) caused by different frequencies of GNSS signals at the receiver. The receiver delay and IFB can be absolutely calibrated by the GNSS signal simulator and high-speed oscilloscope [28,29,30].

The frequency of the GNSS monitoring receiver clock is locked to the UTC(NTSC) 10 MHz frequency signal. The phase of the receiver clock is synchronized with the UTC(NTSC) 1 PPS signal when it is initially powered. During receiver operation, the UTC(NTSC) 10 MHz frequency is always synchronized with the 1 PPS within 1 ns. With such a receiver clock control strategy, the stabilities of the receiver clock and pseudo-range measurement can be guaranteed. Thus, the time offset UTC(NTSC)–UTC_GNSS_ is obtained precisely.

#### 2.2.3. Data Processing Strategy of GNSS Time Transfer Accuracy Assessment

The real-time data of the time offset UTC(NTSC)–UTC_GNSS_ are seriously contaminated by various sources error or noise including the satellite clock correction error in the space segment, ionosphere delay correction error and tropospheric delay correction error in the transmission segment, and receiver measurement noises [28]. Therefore, it is necessary to correct errors in the pseudo-range measurement. Then, data smoothing processing is applied in order to obtain accuracy offset UTC(NTSC)–UTC_GNSS_ with 1 day time interval. Furthermore, the time transfer error sequence UTC_GNSS_–UTC_true_ is calculated according to Equations (2)–(5). Finally, we calculate the statistics of offset UTC_GNSS_–UTC_true_, including the 95th percentile level (95%), the root-mean-square error (RMS), average (AVG), standard deviation (STD), and maximum (MAX). The calculation formula of RMS is as follows:

(6) $$RMS=\sqrt{\frac{\sum_{i=1}^{N}{\left(\Delta UTC\right)}^{2}}{N}},$$

(7) $$STD=\sqrt{\frac{\sum_{i=1}^{N}{\left(\Delta UTC-\bar{\Delta UTC}\right)}^{2}}{N}},$$

where N is the number of samples; i = 1, 2…, N, ΔUTC is time transfer error sequence, and $\bar{\Delta UTC}$ is the mean of the time transfer error sequence.

### 2.3. Uncertainty Budget

There are many kinds of errors during assessment of GNSS time transfer accuracy, including three parts: (1) the uncertainty from broadcast UTCO which is recorded as $\sigma_{UTCO}$; (2) uncertainties related to satellite, signal propagation path, and receiver which are recorded as $\sigma_{GNSST}$; (3) the uncertainty introduced by BIPM which is recorded as $\sigma_{BIPM}$. These factors are not related; hence, the uncertainty components are independent. According to the principle of error analysis, the uncertainty of the results of GNSS time transfer accuracy assessment, recorded as σ, is calculated as follows:

(8) $$\sigma =\sqrt{\sigma_{GNSST}^{2}+\sigma_{UTCO}^{2}+\sigma_{BIPM}^{2}}.$$

In Equation (7), $\sigma_{UTCO}$ can be obtained from the provider’s annual report, $\sigma_{BIPM}$ can be obtained from BIPM, and $\sigma_{GNSST}$ can be obtained by estimating the user equivalent range error (UERE) and time dilution of precision (TDOP), yielding the following formula [9,10,31].

(9) $$\sigma_{GNSST}=UERE\times TODP÷C,$$

where C is the speed of light in m/s; TDOP can be replaced by the time transfer dilution of precision (TTDOP). TTDOP is related to the number of available satellites, about $1/\sqrt{N}$.

UERE is composed of two parts: the signal-in-space range error (SISE) which is associated with satellite clock error and orbit error, and the user equipment error (UEE) introduced by all residual error contributions in the range domain that are not under the direct control of the GNSS system. Considering the case that SISE and UEE are independent, UERE can be calculated by Equation (10), and the UERE evaluation is shown in Table 1 [9,10,32,33].

(10) $$UERE=\sqrt{SISE^{2}+UEE^{2}}.$$

Because of the open environment in which the antenna of the monitoring receiver is mounted, the number of available satellites is 10 for BDS, eight for GPS, six for GLONASS, and sic for Galileo. The uncertainty budget for the assessment results of GNSS time transfer accuracy is shown in Table 2. For GPS and GLONASS, UTCr–UTC(k) refers to UTCr–UTC(USNO) and UTCr–UTC(SU) respectively.

It can be seen from Table 1 and Table 2 that the uncertainty budget of GLONASS time transfer accuracy assessment is relatively large, which is due to the satellite clock error and orbit error. The $\sigma_{UTCO}$ of BDS is large because the method of BDT traceability is indirect. First, the physical signal of BDT is controlled to the local physical reference. Then, BDT connects with UTC composed of two parts: one is the comparison link between the local physical reference and UTC(NTSC), while the other is the international network of time links between UTC(NTSC) and UTC. The uncertainty of BDT traceability is greater than that of other GNSSTs [28].

## 3. Assessment Result and Analysis

The receiver time offset UTC(NTSC)–UTC_GNSS_ was generated at NTSC with 1 day time interval, involving BDS, GPS, GLONASS, and Galileo, in 2021.

**Figure 3:**
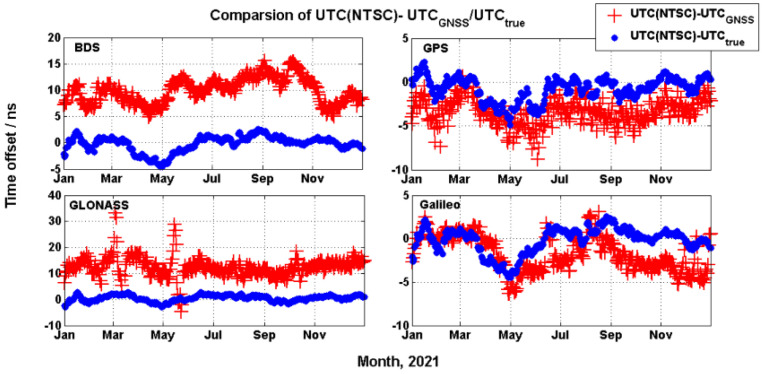
Comparison UTC(NTSC)-UTC_GNSS_ with UTC(NTSC)-UTC_true_ from GNSS.

**Figure 4:**
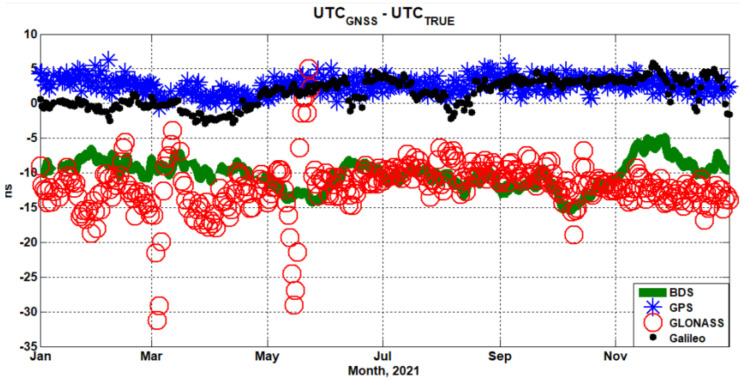
The comparison time transfer error UTC_GNSS_-UTC_true_ for BDS, GPS, GLONASS, and Galileo.

Figure 3 shows the comparison the time offset UTC(NTSC)–UTC_GNSS_ with UTC(NTSC)–UTC_true_. The red plus represents UTC(NTSC)–UTC_GNSS_, while the blue point represents UTC(NTSC)–UTC_true_. Figure 4 shows the comparison time transfer error sequence UTC_GNSS_–UTC_true_ for BDS, GPS, GLONASS, and Galileo. Some conclusions can be drawn from Figure 3 and Figure 4. During the assessment period, the time offset UTC(NTSC)–UTC_GNSS_ of GLONASS had large fluctuations, caused by the fluctuation of UTCO from the GLONASS navigation message. There was a fixed time offset between UTC(NTSC)–UTC_GNSS_ and UTC(NTSC)–UTC_true_ of BDS and GLONASS, which did not exist in GPS and Galileo. The offset reflects the accuracy of UTC_GNSS_ relative to UTC_true_, which is not caused by the GNSS user. The fluctuation range of GLONASS time transfer error was −35 ns to 5 ns, while the fluctuation ranges of BDS, GPS, and Galileo were less than 10 ns.

Table 3 shows the assessment results of time transfer accuracy from BDS, GPS, GLONASS, and Galileo, including 95%, RMS, AVG, STD, and MAX. For BDS, GPS, GLONASS, and Galileo, the time transfer accuracy (with 95%) was 13.8 ns, 4.5 ns, 16.8 ns, and 4.2 ns, respectively. Galileo’s time transfer accuracy was the best, while that of GLONASS was the worst. The AVG values of BDS and GLONASS results reflect the fixed time offset in Figure 3 and Figure 4, which was about 10 ns. However, the STD of the GLONASS time transfer accuracy was the largest, about 3.6 ns.

**Figure 5:**
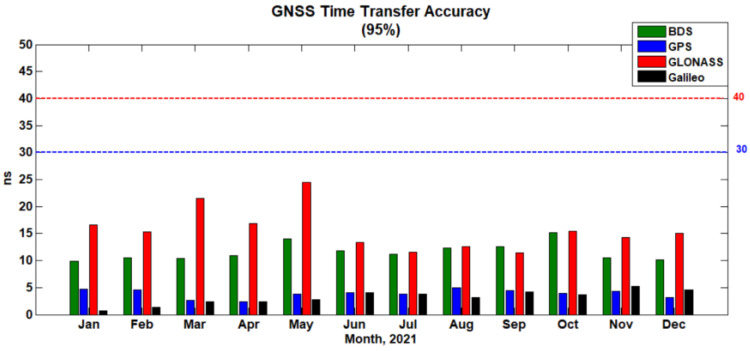
Histogram of monthly GNSS time transfer accuracy assessment results in 2021.

Figure 5 shows the time transfer accuracy of BDS, GPS, GLONASS, and Galileo per month in 2021 (with 95%). In Figure 5, the assessment results of BDS, GPS, GLONASS, and Galileo are expressed in green, blue, red, and black, respectively. The red dashed line indicates that the time transfer accuracy requirement in GLONASS was 40 ns with open service performance specification, while the blue one indicates that it was 30 ns in GPS. The results shown in Figure 5 reveal that the monthly time transfer accuracy met the requirements of GNSS timing performance. The time transfer accuracy of GPS and Galileo was less than 5 ns, that of BDS was between 9 ns and 15 ns, and that of GLONASS was between 11 ns and 25 ns. The BDS and GLONASS timing performance can still be further improved. These conclusions are consistent with the results in Table 3.

## 4. Conclusions

With GNSS modernization, high-precision GNSS timing services are provided for global users, and the timing performance has been widely evaluated. Through a study of the GNSS timing principle, the assessment indicators of GNSS timing performance were discussed, and an assessment method was proposed. The time transfer accuracy of BDS, GPS, GLONASS, and Galileo was assessed and compared. The results allowed the following conclusions to be drawn:

1. The time transfer accuracy refers to the 95% probability level of the instantaneous differences between the estimated receiver time offset from a reference realization of UTC and the true offset over a specified time interval. According to the GNSS time principle, time transfer accuracy is considered as the key GNSS timing performance assessment indicator.
2. An assessment method of time transfer accuracy was proposed on the basis of UTC(NTSC) as an intermediate timescale and UTC/UTC(k) as a reference timescale, and the uncertainty budget was determined. If the local reference time of GNSS users can connect with UTC, this assessment method can be used.
3. The assessment results show that the time transfer accuracy of BDS, GPS, GLONASS, and Galileo was 13.8 ns, 4.5 ns, 16.8 ns, and 4.2 ns respectively, meeting their performance requirements. Specifically, the time transfer accuracy of Galileo was the best, while that of GLONASS was the worst. Furthermore, the time transfer accuracy of GPS and Galileo was much better than their requirement, while the time transfer accuracy of BDS and GLONASS showed a fixed time offset and can still be improved further.

## Figures and Tables

**Table 1:** UERE evaluation.

GNSS	BDS	GPS	GLONASS	Galileo
SISE (m)	0.7	1.0	2.3	0.8
UEE (m)	1.6	1.6	1.6	1.6
UERE (m)	1.7	1.9	2.8	1.8

**Table 2:** The uncertainty budget for GNSS time transfer accuracy assessment.

GNSS		BDS	GPS	GLONASS	Galileo
$\sigma_{GNSST}$ (ns)		1.8	2.2	3.8	2.4
$\sigma_{UTCO}$ (ns)		3.0	1.3	1.7	0.5
$\sigma_{BIPM}$ (ns)	UTCr–UTC(NTSC)	0.7	0.7	0.7	0.7
	UTCr–UTC(k)	/	0.2	0.4	/
σ (ns)		3.6	2.7	4.2	2.5

**Table 3:** The assessment results of GNSS time transfer accuracy.

	BDS	GPS	GLONASS	Galileo
95% (ns)	13.8	4.5	16.8	4.2
AVG (ns)	−10.2	2.6	−11.9	1.6
STD (ns)	2.0	1.2	3.6	2.0
MAX (ns)	15.4	6.3	31.2	5.8
RMS (ns)	10.4	2.9	12.5	2.5

## Data Availability

Not applicable.
